# Olfactory Performance Is Predicted by Individual Sex-Atypicality, but Not Sexual Orientation

**DOI:** 10.1371/journal.pone.0080234

**Published:** 2013-11-07

**Authors:** Lenka Nováková, Jaroslava Varella Valentová, Jan Havlíček

**Affiliations:** 1 Department of Anthropology, Faculty of Humanities, Charles University, Prague, Czech Republic, United States of America; 2 Centre for Theoretical Study, Charles University and the Academy of Sciences of the CzechRepublic, Prague, Czech Republic, United States of America; 3 Department of Zoology, Faculty of Science, Charles University, Prague, Czech Republic, United States of America; Hospital Nacional de Parapléjicos - SESCAM, Spain

## Abstract

Many previous studies have reported robust sex differences in olfactory perception. However, both men and women can be expected to vary in the degree to which they exhibit olfactory performance considered typical of their own or the opposite sex. Sex-atypicality is often described in terms of childhood gender nonconformity, which, however, is not a perfect correlate of non-heterosexual orientation. Here we explored intrasexual variability in psychophysical olfactory performance in a sample of 156 individuals (83 non-heterosexual) and found the lowest odor identification scores in heterosexual men. However, when childhood gender nonconformity was entered in the model along with sexual orientation, better odor identification scores were exhibited by gender-nonconforming men, and greater olfactory sensitivity by gender-conforming women, irrespective of their sexual orientation. Thus, sex-atypicality, but not sexual orientation predicts olfactory performance, and we propose that this might not be limited to olfaction, but represent a more general phenomenon.

## Introduction

 Numerous recent studies have reported sex differences in personality characteristics, cognition, and behavior [1-3]. For instance, robust sex differences have been repeatedly found in physical aggression, which is on average higher in males [4], and in empathy, in which females typically score higher than males [5]. Furthermore, some of these sex differences seem to appear at least as early as during infancy and preschool age, as suggested, for instance, by studies on sex specificity in childhood play behavior [6]. Some of the sex-related differences have also been documented in heterosexual and non-heterosexual individuals. Specifically, it has been shown that, on average, homosexual men tend to show several sex-atypical, i.e. feminine, psychological characteristics. For example, it has been reported that homosexual men exhibit higher empathy and lower physical aggressiveness than heterosexual men [7]. Also, homosexual men outperform their heterosexual counterparts in verbal associations, while the opposite pattern has been found in spatial abilities, particularly in mental rotations [8].

 It has been suggested that many sex differences in psychology develop under the influence of context-dependent epigenetic factors. One such factor largely determining sex differences is prenatal or early perinatal exposure to androgen steroids, which affect sex differences in brain anatomy, and consequently sex differences in behavior, cognition, personality factors, and others [9,10]. Numerous neuroanatomical differences between men and women have been described, such as those in the percentage and asymmetry of the principal cranial tissue volume, which were found to correlate with cognitive performance [11], or synaptic organization of the medial amygdala, which is hypothesized to provide a sexually dimorphic neural substrate for the effects of hormones on adult social behavior [12]. A well-established example of the linkage between a brain region and sexual behavior is the Third Interstitial Nucleus of the Anterior Hypothalamus (INAH-3), which is generally larger in males than in females [13]. Interestingly, this structure is also larger in heterosexual men than in homosexual ones [14]. 

Besides differences in neuroanatomy, prenatal hormonal influences on personality and behavioral sex differences have been studied indirectly via physical traits, which also develop in utero under the influence of steroid hormones and remain stable across the lifespan. In particular, the ratio between the second and fourth digit (2D:4D) is considered a marker of prenatal androgen influence [15]. It develops prenatally [16], seems unaffected by postnatal variations in androgen levels [15], and several studies have reported a higher 2D:4D in females [17], but see 15. In homosexual men, sex-atypical 2D:4D has also been demonstrated [18], but see 19.

 Furthermore, it has been suggested that similar mechanisms that are supposed to influence the average differences between men and women also give rise to intrasexual variation in such traits [20]. Thus, both men and women vary in the level of development of traits which are typical of their own or the opposite sex and, consequently, both men and women can show rather sex-typical or sex-atypical psychological characteristics [20]. It is worth pointing out that despite an association between sexual orientation and psychological sex-atypicality, which is often described in terms of childhood gender nonconformity, empirical evidence suggests that childhood gender nonconformity is not a perfect correlate of non-heterosexual orientation in adulthood since only a proportion of homosexual individuals show sex-atypical traits. For example, about a third of gay men recalled childhood gender-conforming behavior similar to that of heterosexual men [21]. Also, some studies have failed to replicate the previous results on the relationship between sexual orientation and sex-related traits such as 2D:4D [19] or cognition [22]. Consequently, some of the reported differences between heterosexual and non-heterosexual individuals thus might rather represent an epiphenomenon of variability in childhood gender nonconformity.

 To test the spurious association between gender nonconformity and sexual orientation, we singled out olfactory abilities, which tend to exhibit significant sex differences in favor of women [23], especially as regards the ability of odor identification. It is established that performance on this particular test is affected by cognitive factors such as verbal abilities and verbal fluency in particular, in which female superiority has been widely reported [24]. Nevertheless, differences in verbal fluency related to sexual orientation have also been demonstrated, with gay men tending to score the highest or similarly to heterosexual women and lesbian women scoring the lowest or similarly to heterosexual men [25]. Thus, there are reasons to expect similar differences related to sexual orientation in odor identification. However, at the same time, the authors could not demonstrate clear superiority of heterosexual women over heterosexual men on all the three tests of verbal fluency employed. This might indicate the involvement of sex-atypicality rather than sexual orientation in similar tasks. 

The aim of the present study was to explore interindividual differences in olfactory performance related to sex-atypicality, which is often described in terms of childhood gender nonconformity (CGN), and sexual orientation. We expected that, irrespective of their sexual orientation, men exhibiting lower CGN scores, who were more gender-conforming in childhood, would be outperformed by their less gender-conforming counterparts on the test of odor identification, whose scores would resemble those of the more gender-conforming women. Odor discrimination and the olfactory threshold, in which sex differences are less pronounced, should be less likely to produce such results. 

## Materials and Methods

### 1: Ethics Statement

The study complies with the Declaration of Helsinki for Medical Research involving Human Subjects and was approved by the IRB of the Faculty of Science of Charles University. The participants provided written informed consent and received a reimbursement of CZK 300 (approximately US$  15).

### 2: Participants

The sample comprised 156 university students or alumni (67 female and 89 male; mean age 24.2 ± 4.1; range 19 - 35 years). They were recruited by means of snowball sampling from students enrolled on undergraduate and graduate courses lectured by LN and JVV. Furthermore, members of the university's student sexual minority association “Charlie” were invited to participate. Both male and female participants were recruited with regard to their declared sexual orientation (heterosexual/non-heterosexual) to obtain four comparable samples of male and female heterosexuals and non-heterosexuals, respectively. Therefore, in order to be able to perform meaningful comparisons on the four groups, the present sample purposely did not reflect estimated frequencies of non-heterosexually oriented individuals in the general population [26]. To avoid systematic differences in hormonal contraceptive use between heterosexual and non-heterosexual women that might affect olfactory perception, only non-users were recruited.

### 3: Questionnaires

**3.1. General Demographics**

For each participant, data on age, socioeconomic status, religious beliefs, smoking and substance use history, living environment pollution, history of olfaction-related health issues including chronic neurological disorders, head injury, and respiratory allergies and, in women, average length of the menstrual cycle and the start date of the last menstrual period were collected. Day count method was employed to roughly estimate the participants’ menstrual cycle phase at the time of testing. There were no sex differences in age, Mann-Whitney U = 2769.5, p = .52. The vast majority of participants (90%) were non-smokers, with smokers (N = 16, 8 males) reporting 1.88 ± 1.59 pack-years (range 0 - 6) and exhibiting no differences with regard to sex or sexual orientation in lifetime tobacco exposure. There were no reports of substance use, chronic neurological disorders, head injuries or respiratory allergies. All women were regularly cycling and reported a usual menstrual cycle length of 29 ± 3 days (range 26 - 33 days). Menstrual cycle phase at the time of testing was random across participants and therefore not controlled for in any of the analyses.

**3.2. Sexual orientation assessment (The Kinsey Scale**)

All participants indicated their sexual orientation on the Kinsey Scale [27], prompted by the statement “I regard myself as…”. The seven-point ordinal Kinsey Scale, ranging from zero to six, was anchored on both ends, with zero labeled “heterosexual” and six labeled “homosexual”. It is important to note differences in sexual orientation between men and women. There is a robust body of evidence suggesting greater fluidity in women’s sexual orientation compared to that of men, particularly as regards non-heterosexual women [28]. Female non-heterosexuality is significantly less stable than heterosexuality, whilst in men, both heterosexuality and homosexuality are relatively stable [29]. Also, women are more likely than men to use the middle categories of the Kinsey scale to indicate their sexual orientation [30]. Thus, to handle the resulting problem of necessarily different distributions of sexual orientation categories in men and women, we followed an approach previously adopted by some authors, e. g. Santtila and colleagues [31], and for the purposes of the analysis of variance performed on the total sample, the categories were merged to produce the following groups: the “heterosexual” group (N = 73, 41 males), comprised of individuals who considered themselves exclusively heterosexual (ratings of “0”) or predominantly heterosexual, only incidentally homosexual (ratings of “1”) and the “non-heterosexual” group (ratings of „2“ to „6“; N = 83, 48 males). This approach afforded meaningful comparisons performed on the total sample among similar groups, while accommodating the fact that women (but not men) tend to identify themselves as predominantly, rather than exclusively heterosexual or homosexual [32,33]. The groups did not differ in terms of age, F(3,151) = .806, p = .49. Please see Table 1 for frequency counts and percentages of sexual orientation categories in men and women.

**Table 1:** Frequency counts and percentages of sexual orientation categories in men (N=89) and women (N=67), respectively, and sex difference between relative percentages (two-sided).

**Sexual orientation category**	**Count**		**Percent**		**Percentage difference**
	M	F	M	F	
0	29	15	32.58%	22.39%	p = .16
1	12	17	13.48%	25.37%	p = .06
2	1	11	1.12%	16.42%	**p < .001**
3	2	4	2.24%	5.97%	p = .23
4	3	2	3.37%	2.99%	p = .89
5	10	7	11.24%	10.45%	p = .88
6	32	11	35.96%	16.42%	**p < .01**

**3.3. Childhood Gender Nonconformity**

To retrospectively assess the participants’ childhood sex-typed behavior and gender identity, the participants were administered a sex-appropriate form of the Czech version of the Childhood Gender Nonconformity Scale (CGN, [34]). The scale consists of seven items rated on a 7-point Likert scale, anchored on both ends with “strongly disagree” (1) and “strongly agree” (7), respectively. Items cover internal feelings of maleness or femaleness (“As a child I often felt that I had more in common with girls/boys than boys/girls.”) and participation in sex-stereotypic games and activities (“As a child I (dis)liked competitive sports such as football, baseball, and basketball.”). Scores on individual items were added up to produce the overall score, which can range between 7 and 49, with higher scores indicating greater gender nonconformity.

**3.4. Continuous Gender Identity**

To assess the participants’ current self-concepts as masculine or feminine, a sex-appropriate form of the Czech version of the Continuous Gender Identity Scale (CGI; [34]) was administered. The measure includes 10 items rated on a 7-point Likert Scale ranging from “strongly disagree” (1) to “strongly agree” (7) which relate to how masculine or feminine the participant feels (“In many ways I feel more similar to men/women than to women/men.“) and behaves (“People think I should act more feminine/masculine than I do.“). Scores on the individual items are added up to produce the overall score, which can range between 10 and 70. The more masculine a woman’s self-concept is, the higher the score, whereas men scoring high on the CGI tend towards more feminine self-concepts. Both the CGN and CGI were translated to the Czech language by JVV and back translation was produced by LN.

Since this study was part of a broader project, including a study by Havlíček and colleagues [35] on the relation of Big Five personality traits and olfactory abilities, the participants further completed several other questionnaires.

### 4: Olfactory measures

The Sniffin’ Sticks test [36], manufactured by Burghart Messtechnik GmbH, was used to obtain all olfactory measures. This is one of the most widely used tests of (ortho)nasal chemosensory performance, based on pen-like odor dispensing devices. The extended version of the test is comprised of three tests of olfactory function, namely odor threshold (olfactory sensitivity), discrimination, and identification.

The olfactory threshold refers to the minimum concentration of a tested odorant (n-butanol) that an individual is able to reliably differentiate from a blank sample. The set consists of 16 dilution steps of the odorant (targets), each of which forms a triplet with two blanks. A single-staircase, three-alternative forced-choice (3-AFC) method is used, in which, starting with the lowest concentration (dilution number 16), an ascending (low to high concentration) series of even-numbered triplets is presented, with successful trials prompting another presentation of the same triplet in a random order. Two successful trials in a row mark a turning point; starting with the nearest lower concentration, a descending series of triplets is presented until the individual fails to detect the target. This marks a reversal towards the higher concentrations and, starting with the next higher concentration, an ascending series of triplets is presented until two correct trials occur, marking another reversal. The testing is finished after a total of 7 reversals is reached. The threshold score is computed as the arithmetic mean of the dilution number at the last four reversals. Ranging from 1 to 16, higher scores indicate greater olfactory sensitivity (i.e. lower threshold). 

The test of odor discrimination assesses the degree to which an individual can differentiate between odors in suprathreshold concentrations. The set comprises 16 triplets of odorized pens, of which two are identical, and the individual is asked to indicate the odd one. The score is the total of correct trials (0 - 16), with higher scores indicating a better ability of odor discrimination. 

The 16-item test of cued odor identification involves a 4-AFC task in which the individual is required to choose a label from a list of four, which he or she thinks best describes the odor’s source. The score is the total of correct trials. 

Based on the composite score of the three tests (TDI), individuals can be classified as normosmic (intact sense of smell; TDI > 30), hyposmic (TDI 30 - 15), or functionally anosmic (TDI < 15) [36]. Although all participants reported good respiratory health, there were 11 instances of hyposmia in the sample: 10 mild (TDI 25 - 30) and 1 moderate (TDI 20 - 25). The one case of moderate hyposmia was excluded from the analysis because more consequential factors than a mere momentary lapse in olfactory performance were likely involved [36].

### 5: Procedure

Individual, one-per-person testing sessions were conducted by LN in the morning hours or by early afternoon (3 p.m.) in a well-ventilated room. Individuals were instructed to only participate if in good respiratory health and were asked to refrain from smoking or consumption of odorous foods at least 2 hours prior to participation, as well as to forego applying perfume or other scented cosmetic products. The researcher first introduced the procedure, assured the participant the data would be subject to confidential treatment, and provided financial recompense for participation. In winter time, participants were first asked to complete the questionnaires before proceeding to olfactory testing so that their olfactory performance would not be affected by abrupt changes in ambient temperature. Within the olfactory testing part of the session, olfactory sensitivity was always tested first, followed by discrimination and identification. The participants were allowed a three-minute break after each test to prevent olfactory adaptation. The entire session took, in most participants, 75 to 90 minutes.

### 6: Analyses

All analyses were carried out with SPSS 18.0 (IBM Corp.). Data normality was checked firstly by visually examining individual histograms of all relevant variables, secondly by producing skewness and kurtosis values and their respective standard errors, from which z-scores were computed and compared to the value of 1.96, as suggested by Field [37], and thirdly with multiple Shapiro-Wilk's W tests. Since departure from normality in nearly all variables was detected, nonparametric tests were used where possible. 

Differences in CGN and CGI scores related to sex and sexual orientation were analyzed using the Kruskal-Wallis ANOVA. To analyze differences in olfactory measures, we ran a MANCOVA, which is considered to be robust to violations of multivariate normality, as well as to violations of homogeneity of variance/covariance matrices, if N of the largest group is no more than about 1.5 times the N of the smallest group [37], which was met. To look for possible covariate candidates (e.g. age) to include in the analysis, a Kendall correlation matrix was produced. Further, for the categorical predictors of sex and sexual orientation, a point-biserial correlation and a biserial correlation were carried out, respectively. Since the identification score turned out to be positively associated with age (Kendall Tau = .15, p < .01), age was subsequently entered in the MANCOVA as a covariate. Also, the identification score was correlated with both CGN and CGI scores (Kendall Tau = .15, p < .01 and Kendall Tau = .14, p < .05, respectively). However, CGN and CGI scores could not be entered in the analysis as covariates given their significant association with both of the dichotomous predictors, sex, r_pb_ = -.34, p < .0001 (both CGN and CGI), and sexual orientation, r_b_ = -.45, p < .0001 (CGN) and r_b_ = -.25, p < .01 (CGI), respectively. This was because in instances in which there is nonrandom group assignment and a variable is intimately associated with any of the independent variables so that the groups inherently differ on this variable, use of such a variable as a covariate is incorrect [38]. Nevertheless, the effect of CGN, which is the strongest correlate of adult sexual orientation [21], on prediction of olfactory scores was tested by means of a regression analysis, as detailed below. Finally, there was an association between CGN and CGI scores, Kendall Tau = .46, p < .0001.

The three olfactory scores (threshold, discrimination, identification) were entered in the MANCOVA as dependent variables, sex and sexual orientation as dichotomous categorical factors, and age as a covariate.

 The follow-up to the MANCOVA was twofold, as recommended by Field [37]. Firstly, a stepwise discriminant function analysis and a subsequent canonical analysis were run, and, secondly, a separate ANCOVA on identification scores and ANOVAs on discrimination and threshold scores were performed, further followed up with multiple Mann-Whitney U tests for post-hoc comparisons. 

 To test whether olfactory scores would be predicted by sexual orientation or, rather, by its strongest correlate, CGN, we ran a categorical regression analysis, using the SPSS Optimal Scaling (CATREG) feature. CGI, which was associated with CGN scores, was not included in the analysis to prevent multicollinearity problems. The assumptions of the test were met since the number of valid cases exceeded the number of predictor variables plus one. Because of the differences in the distribution of sexual orientation categories between men and women, as detailed above, the analysis was run separately for each sex. In men, the categories of 2, 3, and 4, which only contained 1, 2, and 3 observations, respectively, were merged. In women, the same was done with categories 3 (N = 4) and 4 (N = 2). The dependent variables of identification, discrimination, and threshold score were treated as numeric measures, and the CGN and sexual orientation as ordinal measures, which were discretized by ranking. A numerical initial configuration was selected, as recommended when no variables are treated as nominal [39]. Multicollinearity did not appear a serious problem, as the two predictors (sexual orientation and CGN) were only found to be moderately associated, Kendall Tau = .49, p < .0001 and Kendall Tau = .35, p < .0001 in men and women, respectively. This was further supported by reviewing the variance inflation factors (VIF), which were nowhere near the value of 10, and the average VIF was not greater than 1, as recommended by Field [37]. Moreover, a parallel analysis with multiple linear regression showed comparable results.

## Results

### 1: Interindividual differences in CGN and CGI scores

The Kruskal-Wallis ANOVA on CGN scores revealed significant differences H(3, 148) = 55.72, p < .0001, namely between heterosexual men, who exhibited the lowest CGN scores, and everyone else (all p*s* < .001), and between heterosexual and non-heterosexual women (p = .02), with the former being more gender-conforming. There was also a difference in CGI scores, H(3, 148) = 25.49, p < .0001, namely between non-heterosexual women, who scored second highest, and the highest-scoring non-heterosexual men (p = .01) as well as the lowest-scoring heterosexual men (p < .0001). Descriptive statistics of CGN and CGI scores are given in Table 2.

**Table 2:** Descriptive statistics of childhood gender nonconformity (CGN), continuous gender identity (CGI), and olfactory scores in heterosexual and non-heterosexual men and women.

	N	**mean ± SD gender nonconformity scores**		**mean ± SD olfactory scores**		
		CGN	CGI	identification	discrimination	threshold
**men**	88	18.35 ± 8.82	25.28 ± 8.22	13.55 ± 1.52	13.28 ± 1.64	8.12 ± 2.52
heterosexual	40	12.55 ± 4.68	22.78 ± 8.40	13.13 ± 1.32	12.88 ± 1.79	7.86 ± 2.82
non-heterosexual	48	23.19 ± 8.56	27.38 ± 7.52	13.90 ± 1.60	13.63 ± 1.44	8.34 ± 2.25
**women**	67	25.32 ± 10.68	31.58 ± 9.44	13.99 ± 1.24	13.28 ± 1.82	8.52 ± 2.02
heterosexual	32	21.32 ± 10.20	28.87 ± 9.56	14.13 ± 1.21	13.47 ± 1.59	8.55 ± 2.02
non-heterosexual	35	29.59 ± 9.60	34.48 ± 8.53	13.86 ± 1.26	13.11 ± 2.03	8.50 ± 2.05

### 2: Differences in olfactory measures

 The MANCOVA on olfactory measures revealed no sex differences, but a significant effect of the covariate age, F(3, 148) = 3.73, p = .013, which was due to its effect on the identification score. However, there was a significant sex*sexual orientation interaction, F(3,148) = 3.00, p = .033. Results of the first part of the twofold follow-up, the stepwise discriminant function analysis followed by a canonical analysis, suggested that discrimination between groups was significant with Sniffin' Sticks identification and discrimination (but not threshold) scores entered in the model (Wilks' Lambda = .90; F(6,300) = 2.63, p < .02), in which, however, only the identification score was a significant contributor, F(3,150) = 3.63, p = .01. The canonical analysis indicated that there was only one significant discriminant function, accounting for 92% of the explained variance, by means of which the most significant and clear discrimination (although rather small in absolute magnitude) could be made between heterosexual males and other participants. To be specific, the lower the identification and, to a lesser extent, the discrimination score on the Sniffin' Sticks test, the more likely it was that such olfactory performance would be exhibited by a heterosexual male. 

The results of the second part of the follow-up were in accordance with this. An ANCOVA with identification as a dependent variable revealed a sex difference F(1,150) = 5.52, p = .02 and a sex*sexual orientation interaction, F(1,150) = 4. 96, p = .027. Post-hoc comparisons showed that heterosexual men scored significantly lower than everyone else, namely than heterosexual women, Mann-Whitney U = 389, N = 72, p < .005, non-heterosexual men, Mann-Whitney U = 662, N = 88, p < .01, and non-heterosexual women, Mann-Whitney U = 509, N = 75, p = .04. An ANOVA with discrimination as a dependent variable revealed a sex*sexual orientation interaction, F(1,150) = 4.27, p = .04. This was due to a difference between heterosexual men and their non-heterosexual counterparts, by whom they were outperformed, Mann-Whitney U = 713.5, N = 88, p = .04. Descriptive statistics of olfactory measures are given in Table 2.

### 3: Categorical regression of sexual orientation and CGN scores on olfactory measures

A categorical regression analysis with sexual orientation and CGN revealed that in men, CGN but not sexual orientation significantly predicted odor identification scores, β = .403, F = 7.259, p < .0001. Men who tended towards greater gender nonconformity in childhood exhibited a better ability of odor identification than their more gender-conforming counterparts. CGN thus explained a significant proportion of variance in odor identification scores of men, R^2^ = .231, F(8,87) = 2.960, p < .01. No significant results were found for the other two olfactory measures in men.

**Figure 1:**
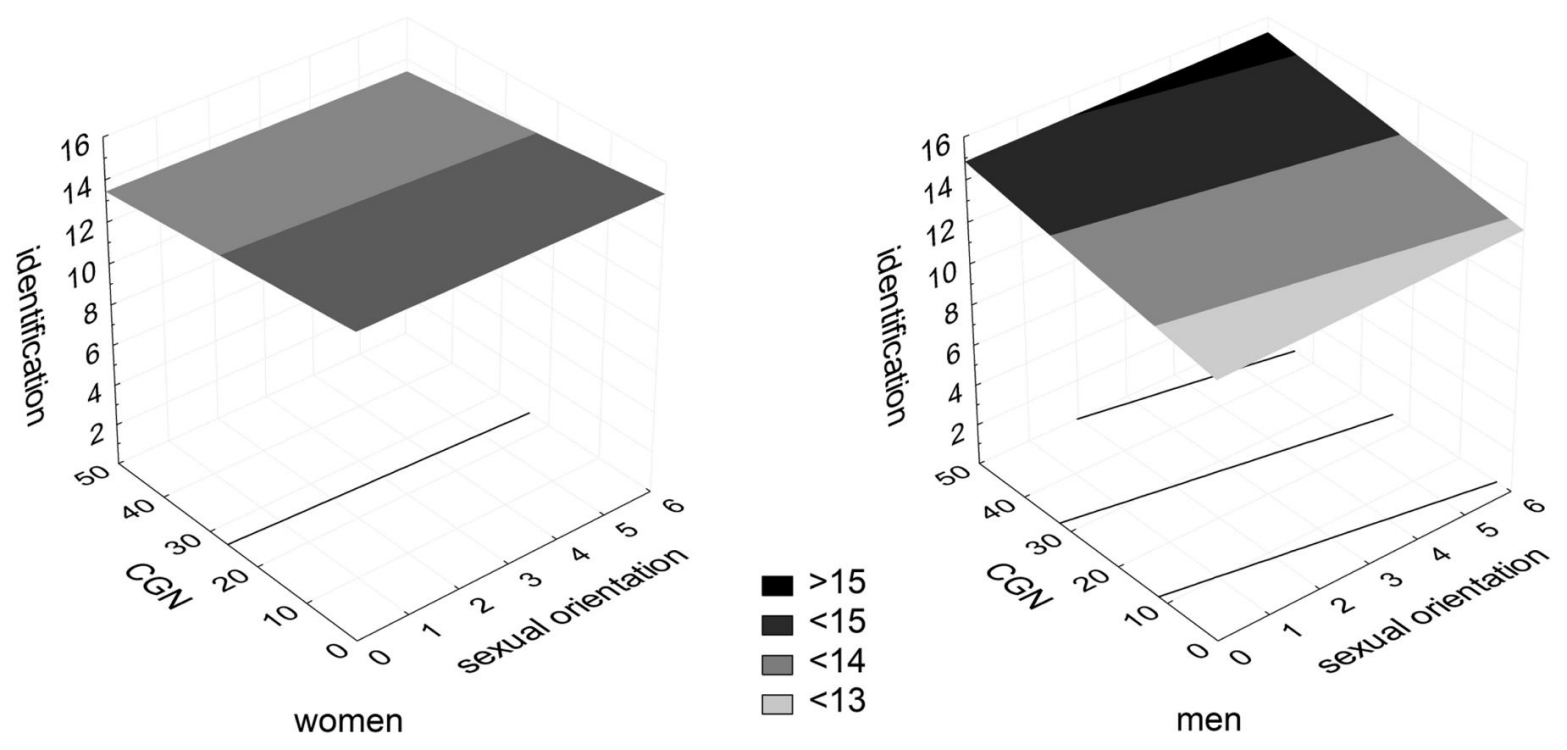
Odor identification scores in men and women relative to CGN and sexual orientation.

**Figure 2:**
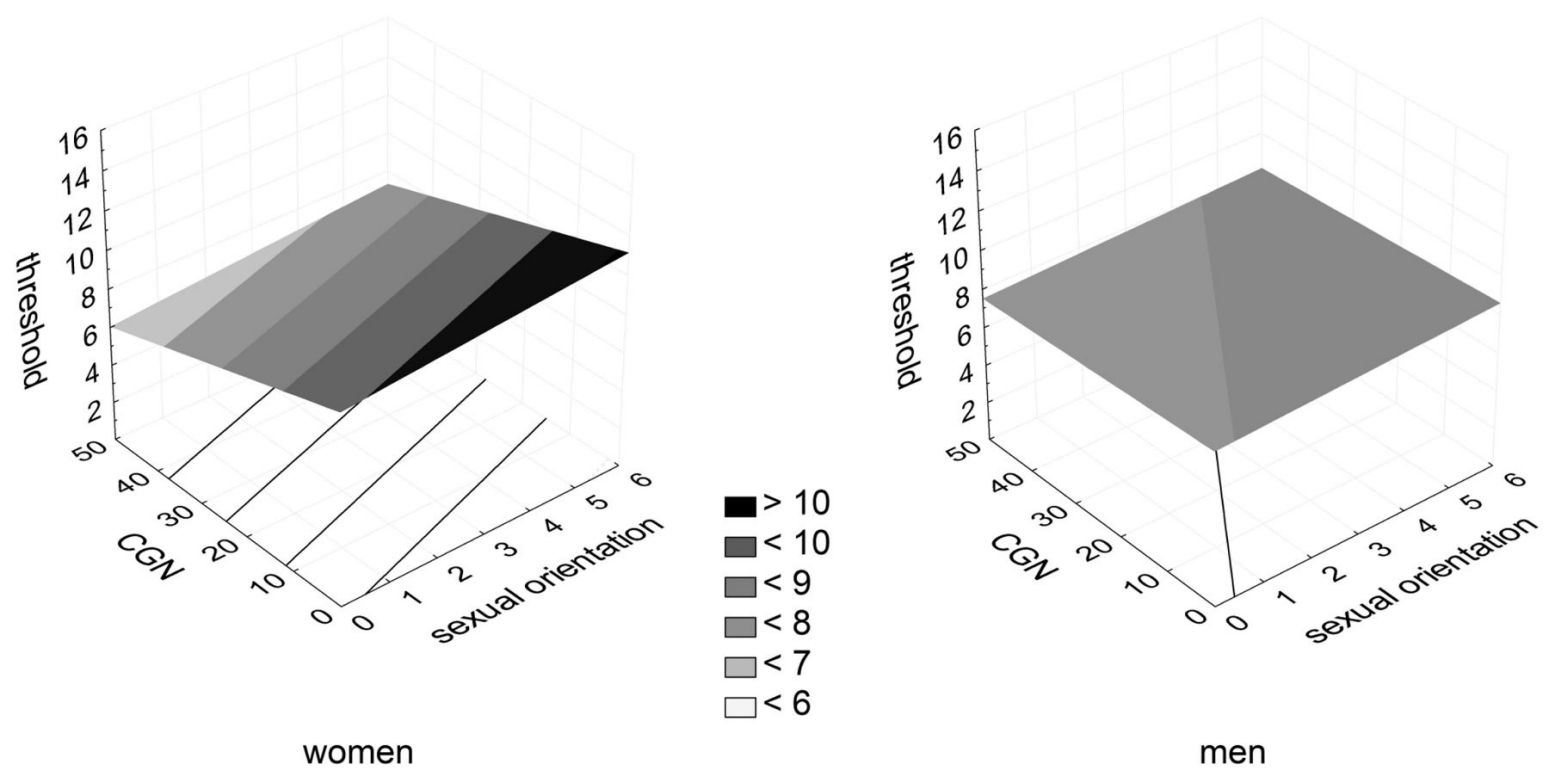
Olfactory threshold scores in men and women relative to CGN and sexual orientation.

In women, CGN but not sexual orientation predicted the olfactory threshold, β = -.569, F = 10.127, p < .0001, suggesting that women who were more gender-conforming in childhood tended to exhibit greater olfactory sensitivity than their less gender-conforming counterparts. However, the overall model was not significant on the conventional level of significance, R^2^ = .247, F(10,59) = 1.607, p = .133. No significant results were found for the other two olfactory measures in women. Odor identification scores and olfactory thresholds in men and women relative to CGN and sexual orientation are plotted in Figures 1 and 2, respectively.

## Discussion

In the present study, we found a modulating effect of sexual orientation on differences between men and women in olfactory performance. Namely, in odor identification, heterosexual men were outperformed by all other participants, and, in odor discrimination, by non-heterosexual men. However, when separate regression analyses were run for each sex in which, along with sexual orientation, CGN was entered as a predictor, only the latter turned out to significantly predict performance on some of the olfactory tests. To be specific, in men, those who had been less gender-conforming in childhood exhibited a better ability of odor identification than the more gender-conforming ones, irrespective of their sexual orientation. In women, those who had been more gender-conforming in childhood exhibited greater olfactory sensitivity than the less gender-conforming ones, irrespective of their sexual orientation. Thus, it would seem that it is CGN rather than sexual orientation that actually modulates differences in olfactory abilities between men and women.

In olfactory research, the number of previous studies which did take into account the possible effect of sexual orientation on interindividual differences in olfaction is very limited. A positron emission tomography (PET) study by Savic and colleagues [40] revealed a sex-dissociated activation of regions covering the sexually dimorphic nuclei of the anterior hypothalamus in response to the putative human pheromones, namely 4,16-androstadien-3-one in women and estra-1,3,5(10),16-tetraen-3-ol in men. This is one of the key brain regions mediating human sexual behavior (e.g. neuroendocrine and autonomic aspects of sexual drive and sexual orientation [41]). In follow-up PET studies, it was found that what actually mattered was not the biological sex but sexual orientation: homosexual men differed from their heterosexual counterparts and resembled heterosexual women in that their preoptic hypothalamus was activated by androstadienone [42]. Similarly, lesbian women, in whom the pattern was less clear, failed to exhibit activation of the region in response to androstadienone, unlike their heterosexual counterparts, but showed some congruence with heterosexual men in their hypothalamic processing of estratetraenol [43]. Nevertheless, a PET study with male-to-female transsexuals [44], whose hypothalamic activation in response to androstadienone and estratetraenol bore some resemblance to that of both heterosexual men and women, indicated that the pattern would likely be more complex. 

By way of explanation, androstadienone is the prominent 16-androstene steroid found in semen, sweat, axillary hair, and blood [45] in much higher concentrations in men than in women, whereas estratetraenol is an estrogen-like steroid reported to be found in the urine of pregnant women [46]. Importantly, some sex-specific effects on the autonomic nervous system as well as mood, memory, and sexual arousal, that act in a context- and dose-dependent manner, have been reported for both substances [47], although the evidence is less consistent for estratetraenol. The above-mentioned sex-specificity of cerebral activation has been interpreted in terms of the supposed bimodality of the stimuli [40,42,43]. However, implicit is the assumption of heterosexual orientation of the participants, i.e. their presumed sexual attraction to the opposite sex, which is the context that lends relevance to interpretations that suggest the pheromone-like nature of these steroid compounds. Nevertheless, several researchers, e.g. Havlíček and colleagues [47], have questioned the ecological validity, and hence the physiological relevance, of androstadienone stimuli employed in the previous studies in the pure crystalline form, and highlighted the critical effect of concentration. What is more, the frequencies of specific anosmias for these compounds in heterosexual and non-heterosexual individuals were not controlled for. Specifically, for androstadienone, women are typically found to exhibit lower thresholds, i.e. greater sensitivity, as shown for instance by Lundström and colleagues [48]. Hence, it is not clear whether heterosexually and non-heterosexually oriented individuals differ in terms of sensitivity and frequencies of specific anosmias for these compounds, which might at least partly account for the pattern of findings reported in these studies.

Thus, the potential effect of sexual orientation on olfactory perception first came to be addressed to help explain findings of sex-dissociated brain activation in response to components of human body odor, i.e. the so-called social odors. The present study, however, aimed to investigate the effect of sexual orientation on the olfactory abilities of odor identification, discrimination, and the olfactory threshold in men and women, tested with odors that are presumed to bear no social relevance. Besides, it should be noted that, in contrast to the aforementioned chemical compounds, frequencies of specific anosmias for the odors employed in the present study are not known but they could perhaps be safely assumed to be substantially lower than those for the androstenes. Although women’s olfactory abilities are often rather simplistically described as being in general superior to those of men, this, in fact, seems to be particularly true for odor identification, in which their olfactory superiority appears to be established relatively early in ontogeny, holds across the lifespan, and exhibits a later decline with aging [49]. It has been argued that the better ability of odor identification in women may be partly accounted for by cognitive factors. It has been found that performance on the test of odor identification is affected by verbal abilities and verbal fluency in particular [49], in which female superiority has been widely reported, e.g. by Halari and colleagues [24]. Moreover, differences in verbal fluency related to sexual orientation have also been demonstrated [25,50], with gay men tending to score higher than heterosexual men or similarly to heterosexual women, and lesbian women scoring lower than their heterosexual counterparts or similarly to heterosexual men. Thus, whether the female advantage in odor identification is driven predominantly by women’s better verbal fluency or not, this should be the primary test in which to look for sexual orientation-related intrasexual differences, with the other two being less likely to produce such results given the less consistent sex differences.

However, in their study, Rahman and colleagues [25] failed to demonstrate clear superiority of heterosexual women over heterosexual men on all the three tests of verbal fluency employed. This might indicate potential involvement of sex-atypicality of individual performance rather than sexual orientation per se. Sex-atypicality is often described in terms of childhood gender nonconformity (CGN), which, mainly in men, is the strongest predictor of sexual orientation in adulthood [21]. Expecting the involvement of CGN, we hypothesized that, irrespective of their sexual orientation, men who were gender-conforming boys would perform primarily in a sex-typical manner and exhibit relatively lower identification scores than those who were gender-nonconforming in childhood and who would be likely to exhibit scores similar to those of gender-conforming women. 

Our data support the expected tendency towards greater CGN in non-heterosexual men and women alike. Also, sex-atypical levels of olfactory performance on the test of odor identification (but not on the other two tests) were found in non-heterosexual men. Heterosexual and non-heterosexual women exhibited no reliable differences in olfactory abilities in the present study. Further, it has also turned out that in men, odor identification scores were actually predicted not by sexual orientation, but by CGN scores. In women, CGN scores rather than sexual orientation appeared to underlie intrasexual variability in the olfactory threshold, although the overall model was not significant on the conventional level of significance.

Given the fact that female olfactory superiority seems to pertain predominantly to odor identification, sex differences in olfaction have often been suggested to be a mere expression of complex differences in higher levels of brain organization and function, e.g. by Schlaepfer and colleagues [51]. If this were the case, the higher scores of women in odor identification could reflect a cognitive advantage that may manifest itself in many other respects. That odor identification and language processing may share some cortical resources has been pointed out for instance by Lorig [52]. In non-heterosexually oriented men, the cognitive pattern (particularly as regards verbal fluency and spatial abilities) was different from that of heterosexual men and not significantly dissimilar from that of heterosexual women [50]. Nonetheless, no such difference was found between heterosexual and non-heterosexual women, who tend to perform primarily in a sex-typical manner [53]. This might explain the absence of significant intrasexual differences in odor identification in women in this study.

In their review, Brand and Millot [23] put forward the hypothesis that women may, in general, encounter olfactory stimuli more often than men and thus they can have greater experience with a wider variety of odors. At least in western industrialized societies, this might be due to women’s long-term greater odor exposure within specific contexts, such as use of cosmetic products or housework [54], which may start as early as in infancy. Gender-nonconforming boys, however, appear to be interested in activities which would be considered typical of the opposite sex, such as doing hair, makeup, dressing-up, cooking or cleaning, as can be gleaned from reports of men who were gender-nonconforming in childhood [55]. Therefore, gender-nonconforming and gender-conforming men (but not women) may, in general, differ in the extent to which they engage in such activities and hence in the level of long-term olfactory experience. In women, intrasexual variability in olfactory abilities was less pronounced. Therefore, it could be speculated that gender-conforming women may not seek more frequent exposure to a significantly wider variety of odors compared to their gender-nonconforming counterparts.

The significance of the present study lies in the finding that CGN rather than sexual orientation underlies intrasexual variability in olfactory abilities. We suggest that this may not be limited to olfaction but in fact represent a more general phenomenon. Several studies have failed to find any sexual orientation-related differences in sex-related traits such as 2D:4D [19], salivary testosterone [8], or certain spatial abilities [22], suggesting that at least some of the reported differences between non-heterosexual and heterosexual individuals might be an epiphenomenon of intrasexual variability in gender nonconformity.

Several studies have recently highlighted the usefulness of quantitative measures of sex-atypicality. The measure of CGN may relate to variability in cognition within and/or between sexual orientation groups, specifically to reading abilities and derived full-scale IQ scores [56] or certain aspects of spatial memory [57]. However, future studies should also test whether the presumed better suitability of CGN for capturing the full range of variability in some traits could not be a mere by-product of the fact that it is measured in a more precise manner than sexual orientation, which can be dichotomous, categorical, or assessed on a seven-point scale at best. 

Finally, a word of caution should be sounded concerning the generalizability of the present findings to the general population. In this study, for the sake of comparability, participants were recruited with regard to their sexual orientation, yielding a ratio of non-heterosexually oriented individuals to heterosexually oriented ones that approached 1:1 within either sex. However, clearly the estimated frequency of non-heterosexually oriented individuals in the general population is much lower [26]. Hence, in order to investigate whether intrasexual variability might interfere with sex differences in psychophysical olfactory performance depending on the number of participants, various samples should be employed in future studies in which the ratio of heterosexual to non-heterosexual individuals would be closer to that typically found in the general population. 

## Conclusions

In conclusion, the present study accentuates the need to employ more comprehensive quantitative measures of sex-atypicality that are known to covary with sexual orientation, such as CGN, to acknowledge the full range of intrasexual variability in traits in which sex differences have been reported. In the present case of olfactory abilities, in which marked differences between men and women are typically noted, the variability observed in various measures was not limited to differences between male and female or heterosexual and non-heterosexual participants. The measure of CGN has afforded finer discrimination of individual performance on some tasks on the basis of recalled childhood sex-atypicality. At the same time, this study is, to the best of our knowledge, the first to demonstrate the effect of CGN and sexual orientation on olfactory abilities. 
