# Correction: Olfactory Performance Is Predicted by Individual Sex-Atypicality, but Not Sexual Orientation

**DOI:** 10.1371/annotation/885ab548-ef01-4e8a-b1c1-464f38ec4d79

**Published:** 2014-01-06

**Authors:** Lenka Nováková, Jaroslava Varella Valentová, Jan Havlíček

Affiliations 1, 2 and 3 for all authors are incorrect. The correct affiliation number 1 is: Department of Anthropology, Faculty of Humanities, Charles University, Prague, Czech Republic. The correct affiliation number 2 is: Centre for Theoretical Study, Charles University and the Academy of Sciences of the Czech Republic, Prague, Czech Republic. The correct affiliation number 3 is: Department of Zoology, Faculty of Science, Charles University, Prague, Czech Republic. 

